# Purification and genetic characterization of gassericin E, a novel co-culture inducible bacteriocin from *Lactobacillus gasseri* EV1461 isolated from the vagina of a healthy woman

**DOI:** 10.1186/s12866-016-0663-1

**Published:** 2016-03-12

**Authors:** Antonio Maldonado-Barragán, Belén Caballero-Guerrero, Virginia Martín, José Luis Ruiz-Barba, Juan Miguel Rodríguez

**Affiliations:** Department of Food Biotechnology, Instituto de la Grasa, Consejo Superior de Investigaciones Científicas (CSIC), Sevilla, Spain; Department of Nutrition, Food Science and Food Technology, Complutense University of Madrid, Madrid, Spain; Present address: Department of Food Biotechnology, Instituto de la Grasa (CSIC), Campus Universidad Pablo de Olavide, Edificio 46. Carretera de Utrera, Km 1, 41013 Seville, Spain

**Keywords:** Bacteriocin, Quorum sensing, Gassericin, Three-component regulatory system

## Abstract

**Background:**

*Lactobacillus gasseri* is one of the dominant *Lactobacillus* species in the vaginal ecosystem. Some strains of this species have a high potential for being used as probiotics in order to maintain vaginal homeostasis, since they may confer colonization resistance against pathogens in the vagina by direct inhibition through production of antimicrobial compounds, as bacteriocins. In this work we have studied bacteriocin production of gassericin E (GasE), a novel bacteriocin produced by *L. gasseri* EV1461, a strain isolated from the vagina of a healthy woman, and whose production was shown to be promoted by the presence of certain specific bacteria in co-culture. Biochemical and genetic characterization of this novel bacteriocin are addressed.

**Results:**

We found that the inhibitory spectrum of *L. gasseri* EV1461 was broad, being directed to species both related and non-related to the producing strain. Interestingly, *L. gasseri* EV1461 inhibited the grown of pathogens usually associated with bacterial vaginosis (BV). The antimicrobial activity was due to the production of a novel bacteriocin, gassericin E (GasE). Production of this bacteriocin in broth medium only was achieved at high cell densities. At low cell densities, bacteriocin production ceased and only was restored after the addition of a supernatant from a previous bacteriocin-producing EV1461 culture (autoinduction), or through co-cultivation with several other Gram-positive strains (inducing bacteria). DNA sequence of the GasE locus revealed the presence of two putative operons which could be involved in biosynthesis and immunity of this bacteriocin (*gaeAXI*), and in regulation, transport and processing (*gaePKRTC*). The *gaePKR* encodes a putative three-component regulatory system, involving an autoinducer peptide (GaeP), a histidine protein kinase (GaeK) and a response regulator (GaeR), while the *gaeTC* encodes for an ABC transporter (GaeT) and their accessory protein (GaeC), involved in transport and processing of the bacteriocin. The *gaeAXI*, encodes for the bacteriocin gassericin E (GasE), a putative peptide bacteriocin (GaeX), and their immunity protein (GaeI).

**Conclusions:**

The origin of the strain (vagina of healthy woman) and its ability to produce bacteriocins with inhibitory activity against vaginal pathogens may be an advantage for using *L. gasseri* EV1461 as a probiotic strain to fight and/or prevent bacterial infections as bacterial vaginosis (BV), since it could be better adapted to live and compete into the vaginal environment.

**Electronic supplementary material:**

The online version of this article (doi:10.1186/s12866-016-0663-1) contains supplementary material, which is available to authorized users.

## Background

Members of the genus *Lactobacillus* are widely recognized as the hallmark of the normal or healthy vagina and are thought to play a major role in protecting the vaginal environment from non-indigenous and potentially harmful microorganisms [1–4]. *Lactobacillus gasseri* is a human autochthonous microorganism [5] and one of the dominant *Lactobacillus* species in the vaginal ecosystem, together with *Lactobacillus jensenii*, *Lactobacillus iners* and *Lactobacillus crispatus* [6–8]. Thus, decreasing of lactobacilli titer in the vagina, results in bacterial vaginosis (BV), where the normal flora is replaced by undesirable bacteria, including bacterial pathogens such *Gardnerella vaginalis*, *Atopobium vaginae*, *Mobiluncus curtisii*, *Prevotella bivia*, *Leptotrichia amnionii*, *Eggerthella* spp,, *Sneathia* spp and *Megasphaera* type I spp. [9–11]. *L. gasseri* has been well documented as a commensal of the vaginal mucosa and exhibits a negative correlation with BV [2–4]. Some strains of this species have a high potential for being used as probiotics in order to maintain vaginal homeostasis [12–14]. *L. gasseri* may confer colonization resistance against pathogens in the vagina by displacing them through competitive adhesion [15, 16] or by direct inhibition through production of antimicrobial compounds, including lactic acid, hydrogen peroxide and bacteriocins [12, 17]. Bacteriocins could play a major role to control non-indigenous or pathogenic organisms in the female genitourinary tract [18]. Bacteriocins are ribosomally-synthesized antimicrobial peptides of bacterial origin that are active against a variable spectrum (from narrow to broad) of bacteria. Production of these antimicrobial peptides plays an important role in bacterial competition, allowing survival advantages to the producing strain [19]. Bacteriocins from Gram-positive bacteria are nowadays classified into two major classes [20]: the lantibiotics (class I; bacteriocins containing post-translationaly modified residues) and the non-lantibiotics (class II; bacteriocins with non-modified residues except for the formation of disulfide bridges and circular bacteriocins). Class II is divided into four subgroups: class IIa (pediocin-like bacteriocins), IIb (two-peptide bacteriocins), IIc (cyclic bacteriocins), and IId (linear non-pediocin-like one-peptide bacteriocins).

Currently, four different bacteriocins from *L. gasseri* have been purified and genetically characterized: gassericin T from *L. gasseri* SBT2055 [21], acidocin LF221A (Acd221A) and acidocin LF221B (Acd221B) from *L. gasseri* LF221 [22], and gassericin A from *L. gasseri* LA39 [23]. While gassericin A is a class IIc circular bacteriocin, gassericin T and acidocins LF221A and LF221B have been proposed to belong to class IIb (two-peptide) bacteriocins, although no experimental data supporting this hypothesis has been published, yet.

In this work we describe the biochemical and genetic characterization of gassericin E, a novel bacteriocin produced by a *L. gasseri* strain isolated from the vagina of a healthy woman. Production of this bacteriocin was shown to be promoted by the presence of certain specific bacteria in co-culture with the producer strain. The genetic background showed the presence of a three-component regulatory operon that may be involved in the regulation of the production of this bacteriocin.

## Methods

### Bacterial strains and growth conditions

*Lactobacillus gasseri* EV1461 was isolated from the vagina of a healthy woman (vaginal exudate), while we were searching for bacteriocin-producing lactobacilli species isolated from different human sources. The volunteer gave written informed consent to the protocol (reference B-06/262), which had been approved by the Ethical Committee of Clinical Research of Hospital Clínico San Carlos Madrid (Spain).

Most of the bacterial strains used in this study (Tables 1 and 2) were grown routinely in MRS medium (Oxoid, Basingstoke, Hampshire, England) at 37 °C in aerobic conditions. *Actinomyces neuii*, *Escherichia coli*, *Staphylococcus* spp. and *Streptococcus* spp., were grown in Brain Heart Infusion (BHI) medium (Oxoid) at the same conditions described above. Fastidious organisms were grown in different culture media at 37 °C in anaerobic atmosphere (80 % N_2_ -10 % CO_2_ -10 % H_2_) as follows. *Gardnerella vaginalis* was grown in Casman’s medium base (BD229010; Difco) with 5 % v/v rabbit blood; *Porphyromonas gingivalis*, *Atopobium vaginae*, *Anaerococcus vaginalis* and *Leptotrichia amnionii* were grown in Tryptic Soy Broth Agar (TSB, Difco); *Prevotella bivia* was grown in Modified Reinforced Clostridial Agar Broth Medium (RCA, Oxoid); *Mobiluncus* spp. were grown in Enriched Tryptic Soy Agar (ETSA) medium (ATCC medium 1257). Unless indicated, strains from human sources belonged to our own collection, while strains from other sources belonged to different bacterial collections (Tables 1 and 2). They were maintained as frozen stocks at –80 °C in their respective culture medium plus 20 % (vol/vol) glycerol.

**Table 1:** Inhibitory spectrum of *Lactobacillus gasseri* EV1461 against bacterial strains from human origin

Bacterial species	Strain^a^	Source^b^	Sensitivity^c^
*Actynomyces neuii*	FR1543, P1543	FVM	++
	O281-1, MP144-2	FVM	++
*Actinomyces turicensis*	MS293-61	FVM	++
*Anaerococcus vaginalis*	DSM 7457^T^	DSMZ	+
*Atopobium vaginae*	DSM 15829^T^	DSMZ	+
*Bifidobacterium longum*	H1542	FVM	-
*Candida albicans*	PS09v, PS144v	FVM	-
*Corynebacterium amycolatun/xerosis*	V294IV-1	FVM	++++
*Corynebacterium aurimucosum*	VL123-1	FVM	-
*Corynebacterium freneyi*	V293III-10	FVM	-
*Corynebacterium glucurunolyticum*	MS293-60	FVM	-
*Corynebacterium tuberculostearicum*	MS293-1	FVM	+++
	MP144-3	FVM	++++
*Corynebacterium simulans*	V191-2	FVM	-
*Corynebacterium striatum*	V123-5	FVM	-
*Enterococcus faecalis*	H1441	FVM	++++
	C901, L1443	FVM	+++
	C301, EV1444	FVM	++
	EV1542, LA1442	FVM	++
	LAM43, LV123	FVM	++
	HK223, MA006	FVM	+
	FR1542, P1441a	FVM	+
	C1002, FR1441, L1543	FVM	-
	M1441, M1541,	FVM	-
	SC1442, SC1541	FVM	-
*Enterococcus faecium*	C656	FVM	++
*Enterococcus gallinarum*	HU521	FVM	++
*Enterococcus saccharolyticus*	HU522	FVM	+
*Escherichia coli*	FR1545	FVM	-
*Facklamia hominis*	EV1443	FVM	-
*Gardnerella vaginalis*	PS07v, PS29v, PS64v	FVM	+++++
	PS33v	FVM	+++
	PS37v	FVM	++++
*Lactobacillus fermentum*	Lc40	FVM	++
*Lactobacillus gasseri*	Lc9, Lc23	FVM	++++
	LA2441	FVM	+++
*Lactobacillus paracasei*	C1351, C1352	FVM	++
*Lactobacillus salivarius*	HN6	FVM	-
*Leuconostoc mesenteroides*	C1353	FVM	+++
*Leptotrichia amnionii*	DSM 16630	DSMZ	-
*Mobiluncus mulieris*	DSM 2710, DSM 25311^T^	DSMZ	-
*Mobiluncus curtisii*			
subsp. *curtisii*	DSM 23059^T^	DSMZ	-
subsp. *holmesii*	DSM 21655^T^	DSMZ	-
*Porphyromonas gingivalis*	DSM 20709^T^	DSMZ	++++
*Prevotella bivia*	DSM 20514^T^	DSMZ	++++
*Propionibacterium acnes*	P1544	FVM	+++
*Propionibacterium avium*	H1544b	FVM	-
*Propionibacterium granulosum*	C1441	FVM	-
*Staphylococcus aureus*	PS11v	FVM	++++
	PS88v	FVM	+++
	PS124v	FVM	++
*Staphylococcus caprae*	FR1541a	FVM	+
*Staphylococcus epidermidis*	C1541, EV1441, EV1541	FVM	*-*
	FR1444, FR1541b	FVM	*-*
	L1442, L1544, L1546	FVM	*-*
*Streptococcus agalactiae*	PS79v	FVM	++
	PS72v, PS77v	FVM	+
	V123-1, V144I-1	FVM	-
	DSM 2134^T^	DSMZ	-
*Streptococcus anginosus*	EV1442	FVM	++
	FR1442, L1441, LA1441	FVM	-
*Streptococcus intermedius*	LA1443	FVM	++
*Streptococcus parasanguinis*	L1541	FVM	-

^a, b^ *Abbreviations:* *C* colostrums, *H* faeces, *EV, O, PS and V* vaginal exudates, *FR* rectal frotis, *M* meconium, *MS* semen, *MP* glans, *L* breast milk, *LA* amniotic liquid, *P* skin, *SC* umbilical cord blood, *FVM* Facultad de Veterinaria (Madrid, Spain), *DSMZ* German Collection of Microorganisms and Cell Cultures (Braunschweig, Germany)

^c^Sensitivity to cell-free supernatants of *L. gasseri* EV1461, assayed by the agar drop diffusion test method. Sensitivity scale: + (<10 mm), ++ (10–11 mm), +++ (12–13 mm), and ++++ (14–15 mm), reflecting the degree of sensitivity according to the diameter of the inhibition halo (in brackets); -, resistant

**Table 2:** Inhibitory spectrum of *Lactobacillus gasseri* EV1461 against strains from different sources

Bacterial species	Strain	Source^a^	Sensitivity^b^
*Bacillus cereus*	ATCC 9139	TNO	-
*Enterococcus faecium*	LP6T1a	CIG	-
*Enterococcus faecalis*	EF1	TNO	-
*Lactobacillus acidophilus*	ATCC 4356	TNO	+++
*Lactobacillus bulgaricus*	ATCC 11842	TNO	-
*Lactobacillus casei*	ATCC 334	TNO	-
*Lactobacillus coryniformis*	Q8	FVM	++
*Lactobacillus curvatus*	NCFB 2739	NCDO	++++
*Lactobacillus fermentum*	ATCC 9338	TNO	-
*Lactobacillus helveticus*	ATCC 15009	TNO	-
*Lactobacillus pentosus*	128/2	CIG	-
	CECT 4023	CECT	-
*Lactobacillus plantarum*	CE3	FVM	-
	NCDO 1193	CIT	-
*Lactobacillus salivarius*	NCFB 2747	TNO	-
*Lactobacillus sakei*	NCFB 2714	TNO	++++
*Lactococcus lactis*			
*subsp. cremoris*	MG1363	CIT	-
	CNRZ 117	INRA	-
*subsp. lactis*	IL1403	INRA	-
*Leuconostoc cremoris*	DB1275	TNO	-
*Pediococcus pentosaceus*	FBB63	TNO	+++
	PC1	TNO	-
*Staphylococcus carnosus*	MC1	TNO	-
*Streptococcus thermophilus*	ST20	TNO	-
	ST112	TNO	-

^a^ *Abbreviations:* *CECT* Colección Española de Cultivos Tipo (Universidad de Valencia, Burjasot, Spain), *CIG* Colección Instituto de la Grasa (Sevilla, Spain), *CIT* Cranfield Institute of Technology (UK), *FVM* Facultad de Veterinaria, Universidad Complutense (Madrid, Spain), *INRA* Institut National de la Recherche Agronomique (Jouy-en-Josas, France), *NCDO* National Collection of Dairy Organisms (Reading, UK), *TNO* Nutrition and Food Research (Zeist, The Netherlands)

^b^Sensitivity to cell-free supernatants of *L. gasseri* EV1461, assayed by the agar drop diffusion test method. Sensitivity scale: + (<10 mm), ++ (10–11 mm), +++ (12–13 mm), and ++++ (14–15 mm), reflecting the degree of sensitivity according to the diameter of the inhibition halo (in brackets); -, resistant

### Bacteriocin assays

To check for bacteriocin production on solid medium, a pointed sterile inoculating handle was soaked in overnight broth cultures of the producing strain and then punctured in MRS agar plates. The plates were incubated at 37 °C for 6 h and then were overlaid with 4.5 ml soft agar (MRS plus 0.75 % [w/v] agar) inoculated with ca. 10^5^ CFU/ml of the indicator strains. Plates were further incubated at 37 °C for 16–18 h, and examined for clear halos of inhibition around the punctured cultures.

Bacteriocin activity in cell-free supernatants (CFSs) from stationary-phase broth cultures (16 h at 37 °C) of *L. gasseri* EV1461 was assayed by using the agar drop diffusion test as described previously [24], using the strains listed in Tables 1 and 2 as indicator microorganisms. Bacteriocin activity was quantified by a microtiter plate assay, as described previously [25] using *L. paracasei* C1351 as the indicator strain. One bacteriocin unit (BU) was defined as the amount of bacteriocin active CFS that inhibited the growth of the indicator strain by 50 %, using as a reference the turbidity of control cultures without CFS added. This was expressed as the reciprocal of the highest dilution exhibiting 50 % inhibition of the indicator strain per milliliter (BU/ml).

### Conditional bacteriocin production of *L. gasseri* EV1461

Two or three (low cell density) and 20 to 50 colonies (high cell density) were inoculated into fresh 10-ml MRS broth tubes and incubated at 37 °C. CFS was tested for antimicrobial activity at 6 and 16 h of growth as described above. The minimum number of cells that were necessary to obtain a Bac+ or a Bac- culture was determined by serial dilutions of a 16 h culture of *L. gasseri* EV1461.

Bac+ CFSs from *L. gasseri* EV1461 were tested for their ability to autoinduce bacteriocin production in *L. gasseri* EV1461 Bac- cultures. For autoinduction, 20 μl of Bac+ CFS (containing 1280 BU/ml) were added to fresh MRS broth (1 ml) containing ca. 10^2^ CFU/ml of an overnight culture of *L. gasseri* EV1461 (Bac-), and incubated at 37 °C for 16 h. In control experiments, the same amount of Bac+ CFS was added to 1 ml MRS broth and then the antimicrobial activity was assayed.

Production of bacteriocins by *L. gasseri* EV1461 in co-cultures with the inducer strains *L. gasseri* Lc9, *Lactobacillus pentosus* 128/2, *Lactobacillus plantarum* CE3 and *Propionibacterium avium* H1544 was determined as described previously [26] with some modifications. Briefly, fresh MRS broth was inoculated with an overnight Bac-culture of *L. gasseri* EV1461 (ca. 10^2^ CFU/ml) plus an overnight culture (ca. 10^2^ CFU/ml) of each inducer strain. The mixed cultures were held at 37 °C for 6 h, centrifuged, and the CFSs adjusted to pH 7.0 with 5 N NaOH, filter-sterilized, and finally their inhibitory activity assayed by the agar drop diffusion test, using *Lactobacillus paracasei* C1351 as the indicator strain. In control assays, all of the strains used in the mixed cultures were propagated as pure cultures in their respective media and then assayed for antimicrobial activity as described above.

### Purification of gassericin E

All of the purification steps were carried out at room temperature, and all of the chromatographic equipment and media were purchased from Amersham Biosciences Europe GmbH, Freiburg, Germany. Gassericin E (GasE) was purified from 2-litre cultures as follows. Two 10 ml MRS tubes were inoculated at high cellular densities by picking ca. 100 isolated colonies of a 48-h plate culture of *L. gasseri* EV1461 and incubated at 37 °C for 16 h. Then, two 200-ml MRS bottles were inoculated each one with the 16 h 10 ml *L. gasseri* EV1461 broth cultures, and further incubated at 37 °C for 10 h. Finally, a 2-litre MRS flask was inoculated with the two 200-ml broth cultures of *L. gasseri* EV1461 and further incubated for 16 h. The cells were removed by centrifugation at 10,000 × *g* for 10 min at 4 °C and, then, the bacteriocin was purified from the CFS by the same method described for plantaricin NC8 [24]. Briefly, the CFS was precipitated with ammonium sulfate (75 % of saturation), desalted through PD-10 columns, and consecutively applied to cation-exchange (SP-Sepharose Fast Flow) and hydrophobic-interaction (Octyl-Sepharose CL-4B) columns. Fractions showing bacteriocin activity were applied to reverse-phase chromatography (RPC) in a C2/C18 RPC column (GE Healthcare) coupled to a fast protein liquid chromatography (FPLC) system. The bacteriocin was eluted from the RPC column with a linear gradient of 2-propanol (Merck) in aqueous 0.1 % (v/v) trifluoroacetic acid. Fractions showing inhibitory activity after the RPC-FPLC were pooled and rechromatographed to obtain pure bacteriocin. Purity of bacteriocin fractions were checked by SDS-PAGE as described below.

### SDS-PAGE

During the purification process, the RPC-FPLC eluted fractions of GasE were analyzed in duplicate by Tris-Tricine SDS-PAGE, using an 18 % acrylamide resolving gel [27]. After electrophoresis at 100 mV for 2 h, one gel was silver stained while the other was used to detect the inhibitory activity in an overlay assay as described previously [24]. *L. paracasei* C1351 was used as the indicator strain. The Precision Plus Protein Dual Xtra (Bio-Rad) was used as molecular weight standards.

### N-terminal amino acid sequence and mass spectrometry

The N-terminal amino acid sequences of purified GasE peptide was determined by automated Edman degradation with a Beckman LF3000 sequencer/phenylthiohydantoin amino acid analyzer (System Gold, Beckman, Fullerton, CA). Molecular mass of the peptides was determined by matrix-assisted laser desorption/ionization time of flight mass spectrometry (MALDI-TOF). These analysis were performed by Dr. Silvia Bronsoms (Servei de Proteòmica i Bioinformàtica, Universitat Autònoma, Barcelona, Spain).

### PCR sequencing and location of the gassericin E *locus*

The primers G1-F, GatX-R, GatA-F and GatA-R were designed on the basis of the locus encoding gassericin T of *L. gasseri* SBT2055 (GeneBank accession number AB029612) (Table 3). The primers GT1-F, GT1-R, GT2-F, GT3-F,GT4-F,GT5-F,GT6-F,GT7-F and GT8-R were designed on the basis of the locus encoding gassericin T of *L. gasseri* LA158 (GeneBank accession number AB710328) (Table 3). Total DNA was extracted from *L. gasseri* EV1461 colonies as described previously [28].

**Table 3:** Oligonucleotides used in this study

Name	Sequence (5′-3′)
G1-F	GAAGTAACAAGTGGCTTAGAT
GatX-R	CCTATTACAAACGATATGGC
GatA-F	AACATTGGCTAACATAGTTG
GatA-R	CATGCTATTGGAACATAGTG
GT1-F	TGAAAAATAGTTAATTGATAACTTAAGA
GT1-R	TTGCCCCAATAGCCAA
GT2-F	GGAGCTTTTGCATATTGAG
GT3-F	TTAGTCAGAATCGTCGG
GT4-F	GCAAGATCCAAATGCAC
GT5-F	TTGCGGCGTTGCTT
GT6-F	GCGATACATCAGGCAT
GT7-F	CAACCGCGACTTCAA
GT8-R	AATAGCGGCTGGAATAATAA

Amplification with the primer pair G1-F/GatX-R was carried in 50 μl reaction mixtures containing 2.5 mM Mg Cl_2_, 1× reaction buffer, 200 μM concentrations of each of the deoxynucleotides triphosphates (dNTPs), 1 μM of each of the primers, 1.25 U of *Taq* DNA polymerase (Ecotaq; Ecogen, Barcelona, Spain) and 5-μl of template DNA. Amplification included denaturation at 94 °C for 4 min, followed by 30 cycles of denaturation at 94 °C for 30 s, annealing at 56 °C for 1 min, polymerization at 72 °C for 1 min, and a final polymerization step at 72 °C for 5 min. Amplification with the primer pair GT1-F/GT8-R was carried out in 50-μl reaction mixtures containing 2.5 mM Mg Cl_2_, 1× reaction buffer, 200 μM concentrations of each of the dNTPs, 1 μM of each of the primers, 1.25 U of *Taq* DNA polymerase (Ecotaq; Ecogen, Barcelona, Spain) and 5 μl of template DNA. Amplification included denaturation at 94 °C for 2 min, followed by 10 cycles of denaturation at 94 °C for 10 s, annealing at 57 °C for 30 s, and polymerization at 68 °C for 3:30 min, 20 cycles of denaturation at 94 °C for 10 s, annealing at 57 °C for 30 s and polymerization at 68 °C for 3:30 min plus 20 s/cycle, and a final polymerization step at 68 °C for 7 min. The amplified fragments were excised from 0.7 % agarose gels, purified using the Nucleospin® Extract II kit (Macherey-Nagel, Düren, Germany), and sequenced using the primers described in Table 3 at the Genomics Unit of the Universidad Complutense (Madrid, Spain). DNA sequences were assembled using the Seqman software in the DNASTAR package and deposited in the GenBank database (accession number KR080485).

### DNA and amino acid sequence analysis

Searches for DNA and amino acid similarities in nucleotide and protein databases were done using the Basic Local Alignment Search Tool (BLAST; http://blast.ncbi.nlm.nih.gov/) [29]. Searches for promoter sequences were done with the Neural Network Promoter Prediction web interface (http://www.fruitfly.org/seq_tools/promoter.html) [30]. For detection of Rho-independent terminators, the ARNold Web server (http://rna.igmors.u-psud.fr/toolbox/arnold/) was used [31]. For alignment of the amino acid sequences of the leader and mature peptides of the bacteriocins, the ClustalW2 multiple sequence alignment program was used (http://www.ebi.ac.uk/Tools/msa/clustalw2/) [32]. For physico-chemical analysis of peptides (isoelectric point, molecular weight) the WinPep program was used (available from http://www.ipw.agrl.ethz.ch/~lhennig/winpep.html) [33].

## Results

### Antimicrobial spectrum of *L. gasseri* EV1461

Bac+ CFSs from *L. gasseri* EV1461 broth cultures showed antimicrobial activity against many indicator strains used in this study, including species both related and non-related to the producing strain as shown in Tables 1 and 2.

### Conditional bacteriocin production

This strain showed antimicrobial activity (Bac+) when grown on solid medium. However, after the inoculation of two or three colonies into fresh MRS broth (low cell density), *L. gasseri* EV1461 cultures do no display antimicrobial activity (Bac-). By contrast, after the inoculation of a larger number of colonies (20–50 colonies; high cell density), *L. gasseri* EV1461 showed bacteriocin activity (Bac+) after at least 6 h of growth, maintaining this activity up to 16 h of growth. Thus, at low cellular densities (i.e. when it is inoculated below 10^5^ CFU/ml) the antimicrobial activity produced by *L. gasseri* EV1461 was low or no detectable (0–160 BU/ml) (Table 4). In these diluted Bac- cultures, the Bac+ phenotype was restored when the strain was grown on solid medium as isolated colonies or in broth cultures after the addition of a Bac+ CFS from a previous bacteriocin-producing culture (Table 4). Thus, the addition of a Bac+ CFS from *L. gasseri* EV1461 to *L. gasseri* EV1461 (Bac-) broth cultures resulted in bacteriocin production (2560 BU/ml) (Table 4), indicating the existence of an autoinducing factor in the supernatant. In control experiments, the addition of a Bac+ CFS from *L. gasseri* EV1461 to MRS broth showed no antimicrobial activity.

**Table 4:** Conditional bacteriocin production of *L. gasseri* EV1461

Culture	Activity (BU/ml)^a^
*L. gasseri* EV1461 (Bac-)^b^	0–160
*L. gasseri* EV1461 (Bac+)^c^	1280
*L. gasseri* EV1461 (Bac-)+ CFS EV1461 (Bac+)^d^	2560
*L. gasseri* EV1461 (Bac-)+ *P. avium* H1544	1280
*L. gasseri* EV1461 (Bac-)+ *L. plantarum* CE3	1280
*L. gasseri* EV1461 (Bac-)+ *L. pentosus* 128/2	2560
*L. gasseri* EV1461 (Bac-)+ *L. gasseri* Lc9	1280
*P. avium* H1544	0
*L. plantarum* CE3	0
*L. pentosus* 128/2	0
*L. gasseri* Lc9	0

^a^Bacteriocin units per mililiter

^b^Bacteriocin non-producing *L. gasseri* EV1461 (<10^5^ UFC/ml)

^c^Bacteriocin producing *L. gasseri* EV1461 (>10^5^ UFC/ml)

^d^CFS: cell free supernatant from a Bac+ *L. gasseri* EV1461 culture containing 1280 BU/ml

In addition, when highly diluted *L. gasseri* EV1461 cultures were co-cultured with certain specific Gram-positive bacteria, production of bacteriocin was notably increased (1280–2560 BU/ml) with respect to that displayed by the EV1461 pure cultures (0–160 BU/ml) (Table 4). Thus, mixed cultures of *L. gasseri* EV1461 with *Propionibacterium avium* H1544b, *Lactobacillus plantarum* CE3, *Lactobacillus pentosus* 128/2 or *Lactobacillus gasseri* Lc9 induced (up to 16 times) bacteriocin production in *L. gasseri* EV1461 (Table 4). None of the inducing strains exhibited any antimicrobial activity when they were cultivated as single, pure cultures. The ability of these species to induce bacteriocin production in *L. gasseri* EV1461 was independent of their resistance or sensitivity to the bacteriocin produced (Table 1).

### Purification of gassericin E

**Fig. 1:**
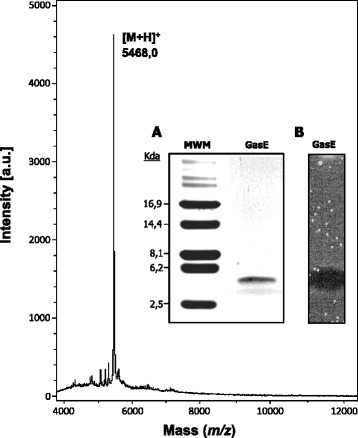
MALDI-TOF mass spectra of purified Gassericin E (GasE). [M+H]^+^, monoisotopic peak of GasE. a.u., absorbance units. Inset panel: SDS-PAGE (A) and bioassay (B) of purified GasE. *L. paracasei* C1351 was used as the indicator strain. MWM, molecular weight marker

GasE was isolated from the Bac+ CFS of a 2-litre broth culture of *L. gasseri* EV1461 as described in the methods section. The behaviour of GasE throughout the purification process was that of a cationic and hydrophobic peptide. Two runs on RPC-FPLC were necessary to obtain fractions containing pure bacteriocin (data not shown). SDS-PAGE analysis showed a single peptide band with an apparent molecular size of 5-kDa (inset panel a in Fig. 1) which presented inhibitory activity against *L. paracasei* C1351 in the corresponding SDS-PAGE activity gel (inset panel b in Fig. 1). The inhibitory activity of pure GasE had a titer of 1280 BU ml^−1^ against *L. paracasei* C1351.

### Mass spectrometry and N-terminal amino acid sequencing

**Fig. 2:**
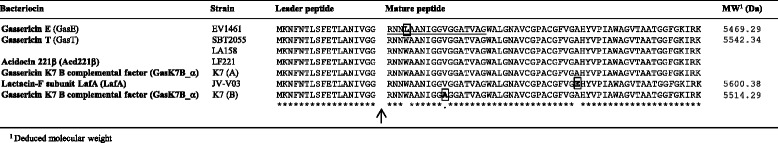
Alignment of the amino acid sequences of double-glycine leader peptides and mature peptides of Gassericin E and other similar gassericin bacteriocins. The sequences were aligned with the ClustalW2 software at the EMBL-Ebi online server. The arrow indicates the Gly-Gly cleavage site of the peptide. Asterisks, dots and double dots mean fully, strongly and weakly conserved residues, respectively. The first 18 aa obtained by Edman sequencing of the peptide GasE are underlined. The two deduced amino acid sequences deposited in databases from *L. gasseri* K7 (A, B) differed in one amino acid at position 11 of mature peptide (VxA). Theoretical molecular weights (MWt) of the mature bacteriocins are shown. The Genebank accession numbers are BAA82353 for Gassericin T, AAP56345 for Acidocin 221B, EFJ70596 for Lactacin-F subunit LafA, AAP73781 and KDA99085for Gassericin K7 B complemental factor (A) and (B), respectively

MALDI-TOF MS analysis of purified GasE indicated that a monoisotopic peak ([M+H]^+^) of the bacteriocin was present, suggesting that the molecular mass of the peptide is 5,468.0 Da (Fig. 1). Partial amino acid sequencing of GasE showed that the 18 N-terminal amino acids shared high similarity with the first 18 N-terminal amino acids of mature gassericin T from *L. gasseri* SBT2055 (Fig. 2).

### DNA and deduced protein sequence analysis of the GasE locus

**Fig. 3:**
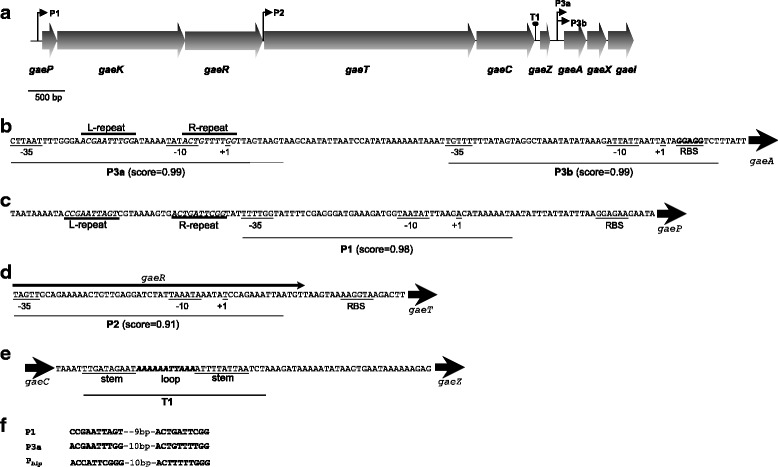
Schematic representation of the locus for Gassericin E (GasE) production (**a**) and detailed analysis of DNA sequences of putative promoters and Rho-independent terminators (**b**, **c**, **d**, **e**, **f**). **a** P1, P2, P3a and P3b are putative promoter sequences, and T1 is a putative Rho-independent transcription terminator. Genes encoding Gassericin E (*gaeA*), the putative complement peptide GaeX (*gaeX*) and their putative immunity protein (*gaeI*), could form an transcriptional unit, driven by two putative alternative promoters (P3a and P3b). Genes encoding the three-component regulatory system formed by the autoinducer peptide (*gaeP*), the histidine protein kinase (*gaeK*) and the response regulator (*gaeR*), as well as the ABC-transporter (*gaeT*) and the accessory protein (*gaeC*), seem to form one transcriptional unit; however two different transcripts could be formed, one larger driven by promoter P1 involving *gaePKRTC* and one shorter driven by promoter P2 involving *gaeTC*. **a**, **b**, **c**, **d** The putative promoters P1, P2, P3a and P3b were detected with the Neural Network Promoter Prediction online server [30], with a promoter score cut-off of 0.9 (score are shown in brackets). The typical -35 and -10 boxes and the ribosome binding sites (RBS) are shown; the +1 indicates the putative transcription start. Putative regulatory DNA sequences (Direct L- and R- repeats) are in italics. **e** Rho-independent terminator; base-pairs are in boldface and apical loop in italics. **f** Alignment of the direct DNA repeats (L and R) found upstream of the GasE and GaeP putative promoters (P1 and P3b) and the consensus L- and R- repeats involved in quorum-sensing regulation of Blp bacteriocins in *Streptococcus thermophilus* [34]

Two DNA fragments of 2.0 and 4.6 kbp were amplified with the primer pairs G1-F/GatX-R and GT1-F/GT8-R, respectively. DNA sequencing of the GasE locus revealed the presence of up to nine open reading frames (ORFs) which seemed to be organized into two putative operons (*gaePKRTC* and *ga*e*AXI*), which could be involved in biosynthesis and immunity of this bacteriocin (*gaeAXI*), and in regulation, transport and processing (*gaePKRTC*) (Fig. 3a). The arrangement of these putative operons was similar to other gene clusters previously described in other bacteriocin-producing *Lactobacillus gasseri* strains (see Additional file 1: Figure S1).

#### GasE biosynthesis and immunity operon

Detailed analysis of the 2 Kbp DNA sequencing of the putative GasE biosynthesis and immunity operon showed that *gaeA* of EV1461 encodes for a peptide of 75 aa which presents an 18 aa double-glycine leader peptide that, upon processing, gives rise to a mature peptide of 57 aa which has a theoretical molecular mass of 5,469.42 Da (Fig. 2). This deduced molecular weight (MW) coincided with the MW of the GasE peptide determined by MALDI-TOF MS (Fig. 1). In addition, the partial 18-aa sequence of GasE obtained by Edman degradation was 100 % identical to the sequence of the first 18 aa deduced of the mature peptide GasE encoded by *gaeA* of *L. gasseri* EV1461. The genes *gatA* from *L. gasseri* SBT2055 and *L. gasseri* LA158, as well as *orfB3 from L. gasseri* LF221 and *L. gasseri* K7, encode for a peptide of 75 aa, which presents a typical double-glycine leader peptide that, once processed, results in a 57 aa mature peptide (gassericin T) with a theoretical molecular mass of 5,542.34 Da (Fig. 2).

Both the amino acid sequence deduced for mature GasE as well as the first 18 aa determined experimentally of purified peptide GasE differed in one amino acid with that deduced for mature GasT. More specifically, GasE contains a leucine residue in position 4 (L4) instead of the tryptophan residue of the peptide GasT at the same position (W4) (Fig. 2).

The leader peptides of GasE of *L. gasseri* EV1461, GasT of *L. gasseri* SBT2055 and *L. gasseri* LA158, Acd221β of *L. gasseri* LF221, GasK7Bα of *L. gasseri*, LafA of *L. gasseri* JV-V03 and lactacin F precursor of *L. gasseri* K7 were 100 % identical (Fig. 3a). However, the hypothetical mature peptide LafA and lactacin F differed in one amino acid with that of gassericin T and gassericin E (Fig. 2).

Immediately downstream of *gaeA*, there were two ORFs (Fig. 3a). The first one (*gaeX*) encodes a putative 65-aa peptide which presents a double-glycine leader peptide that, upon processing, gives rise to a mature peptide of 48 aa and a theoretical molecular mass of 4,763.41 Da. This putative peptide bacteriocin was 100 % identical to GatX of *L. gasseri* SBT2055, LA158, K7, JV-V03 and G7, and 98 % identical to acidocin LF221B and gassericin K7 B of *L. gasseri* LF221 and *L. gasseri* K7, respectively (See Additional file 2: Table S1).

Downstream of *gaeX*, *gaeI* encodes a putative incomplete protein of 88 aa that showed a 100 % of identity with the putative immunity protein to gassericin T of *L. gasseri* SBT2055, LA158, JV-V03, K7, G7 and LF221 (see Additional file 2: Table S1).

Two putative promoter sequences (P3a and P3b) containing the typical -10 and -35 regions were found upstream of *gaeA* thus suggesting that the genes *gaeA*-*gaeX*-*gaeI* are produced on the same transcript (Fig. 3b). In addition, upstream of P3b and overlapping P3a, two imperfect direct repeats of 9 bp separated by a rich AT region were found (Fig. 3b). These putative regulatory elements showed a high similarity with those of *Streptococcus thermophilus* involved in quorum-sensing regulation of Blp bacteriocins [34] (Fig. 3f).

#### Regulatory operon

In *L. gasseri* EV1461, the regulatory operon for bacteriocin production appears to involve three ORFs: *gaeP, gaeK* and *gaeR* (Fig. 3a). The gene *gaeP* encodes a putative peptide of 50 aa residues showing all the features previously described for inducer peptides, known also as autoinducing peptides or peptide pheromones [35, 36]. As with bacteriocin-like peptides, the product of *gaeP* possesses a leader sequence of the double-glycine type that, once processed, gives rise to a mature peptide of 24 aa residues, with a theoretical pI of 11.6 and a MW of 2,905.31 Da. This putative inducer peptide was 100 % identical to that harboured by *L. gasseri* LA158, JV-V03 and K7 (see Additional file 2: Table S1).

The *gene gae*K, which encodes a putative protein showing significant homology with the family of the histidine protein kinases (HPK), was located 2 bp downstream of *gaeP*. The highest similarity for GaeK (99 % identity) was obtained with GatK, the HPK of the gassericin T locus of *L. gasseri* LA158.

Immediately downstream of *gaeK*, the gene *gaeR* was found. The putative protein encoded by *gaeR* was 100 % identical to the response regulators previously found in *L. gasseri* LA158, JV-V03 and K7 (see Additional file 2: Table S1). Therefore, based on homology and relative position, the *gaeP*-*gaeK*-*gaeR* gene cluster seems to form a regulatory operon of the so-called three-component type involved in regulation of bacteriocin production. A similar cluster was found in the genomes of *L. gasseri* LA158 (GenBank acc. num. AB710328), JV-V03 (GenBank acc. num. ACGO02000001) and K7 (GenBank acc. num. ASRG01000003). A putative promoter sequence (P1) containing the typical -10 and -35 regions was found upstream of *gaeP* (Fig. 3c). In addition, upstream of P1, two imperfect direct repeats of 10 bp separated by a rich AT region were found. These regulatory elements were similar to those found upstream of promoter P3a (Fig. 3f).

#### Transport and processing operon

Downstrean of the gene cluster *gaePKR*, the ORF *gaeT* encodes a 719 aa putative protein with 100 % identity with the putative ABC-transporter of gassericin T encoded by *gatT* of *L. gasseri* LA158, JV-V03 and K7 (See Additional file 2: Table S1). Just 1 bp overlapping *gaeT*, *gaeC* encodes a putative 197 aa protein with a 99 % identity to the accessory protein for the ABC-transporter of *L. gasseri* JV-V03, LF221 and K7 (see Additional file 2: Table S1). A putative promoter sequence (P2) was found upstream of *gaeT*, just overlapping *gaeR* (Fig. 3a and d). In addition, two inverted repeats of 10 bp (separated by a rich AT region of 11 bp) which may function as a Rho-independent transcription terminator were found downstream of *gaeC* (Fig. 3e).

The existence of two promoter sequences, P1 and P2, and a terminator (T1) downstream of *gaeC* indicates that two putative transcripts could be formed, one driven by the P1 promoter that would include co-transcription of the *gaeP-gaeK-gaeR-gaeT-gaeC* genes, and one driven by the promoter P2, that would include co-transcription of *gaeT*-*gaeC* genes (Fig. 3a).

## Discussion

In this work, we describe the purification and genetic characterization of gassericin E, a novel bacteriocin produced by *L. gasseri* EV1461, a strain isolated from the vagina of a healthy woman. Gassericin E is very similar to gassericin T, a bacteriocin purified from *L. gasseri* SBT2055, a strain isolated from human feces [21]. In fact, the amino acid sequence of the mature active peptide GasE differs only in one amino acid from that of GasT (Leu4 and Trp4, respectively). In addition, close to the structural gene encoding GasE (*gaeA*), there was a second structural gene (*gaeX*), which encodes a bacteriocin-like peptide (GaeX) 100 % identical to the putative peptide GatX of *L. gasseri* SBT2055 and the gassericin K7 B peptide of *L. gasseri* K7. In contrast, the amino acid sequence of the bacteriocin acidocin LF221 B from *L. gasseri* LF221 is 98 % identical to that of GatX of *L. gasseri* SBT2055; *L. gasseri* LF221 also produces the Acd221β peptide, which is 100 % identical to gassericin T. It is important to note that the putative peptides encoded by *orfB3* from *L. gasseri* LF221 (Acd221β) and *L. gasseri* K7 (GasK7Bα) are 100 % identical to gassericin T but, surprisingly, they have received different names [37, 38]. Recently, the genome sequence of *L. gasseri* K7 (GenBank accession number: ASRG02000002) has shown that the deduced amino acid sequences of GasK7Bα differed in one amino with that previously described [38].

Interestingly, while GasE and GasT were purified from supernatants of *L. gasseri* EV1461 and SBT2055, respectively, the peptides Acd221β and GasK7_α were not detected as active components in the CFSs of *L. gasseri* LF221 and *L. gasseri* K7, respectively. On the other hand, active acidocin LF221 B and GasK7 B were isolated from *L. gasseri* LF221 and *L. gasseri* K7 cultures, respectively, while the GatX and GaeX peptides were not detected in *L. gasseri* SBT2055 and *L. gasseri* EV1461 supernatants, respectively. However, both biochemical and genetic data indicate that the peptide pairs GasE/GaeX, GasT/GatX, Acd221B/Acd221β and GasK7 A/GasK7 B are bacteriocins of the class IIb (two-peptide bacteriocins). Class IIb two-peptide bacteriocins consists of two different peptides whose genes are next to each other in the same operon and whose optimal antibacterial activity requires the presence of both peptides [35]. Thus, on the basis of the genetic organization of the genes involved in GasE biosynthesis this bacteriocin can be considered as belonging to the class IIb two-peptide bacteriocins, being the first component of the bacteriocin. Peptide GaeX is most probably the second component of this two-peptide bacteriocin, although assays of complementary activity between GaeE and GaeX are necessary to confirm this hypothesis.

Interestingly, the inhibitory spectrum of gassericin E appears to be slightly different to that of gassericin K7, a putative two-peptide bacteriocin composed of the peptides K7 A and K7 B, which are virtually identical to GastT and GatX of *L. gasseri* SBT2055, respectively. More specifically, the CFSs of *L. gasseri* EV1461 presented a high inhibitory activity against *L. curvatus* NCFB 2739 and *P. pentosaceus* FBB63 (Table 1) while those of *L. gasseri* K7 had no inhibitory activity against such strains [38].

It has been shown that a single amino acid change in one of the peptides which compose a two-peptide bacteriocin can be responsible for determining the specificity for the target strains, as it is the case of enterocin C (composed by EntC1+EntC2) [39]. The aa sequence of peptide EntC1 was identical to that of Ent1071A, while EntC2 differed only in one amino acid from Ent1071B (Ala17 and Thr17, respectively). This difference was sufficient to explain the different inhibitory spectrum of the bacteriocins enterocin C (EntC1+EntC2) and enterocin 1071 (Ent1071A+Ent1071B) [39].

In fact, although GaeX from CFSs of *L. gasseri* EV1461 has not been purified yet, we observed the existence of putative complementary activity during bacteriocin purification since halos of antimicrobial activity were detected between adjacent drops of assayed fractions, which probably resulted from diffusion and mixing of complementary peptides (see Additional file 3: Figure S2). Recently, during purification of gassericin T from *L. gasseri* LA158, both peptides (GasT and GatX) were detected in active fractions by MALDI-TOF-MS analysis [40].

Production of GasE was lost in highly diluted cultures but it could be restored by the addition of a CFS from a previous Bac+ culture or by the co-culture with other specific Gram-positive bacteria. This fact has been previously observed in other class II bacteriocins whose production is regulated by a quorum sensing mechanism involving a three-component regulatory system (TCRS) consisting of an autoinducer peptide (AIP; known also as inducer peptide or peptide pheromone), a histidine protein kinase (HPK) and a response regulator (RR) [41, 42]. The AIP is secreted to the medium, being sensed by the membrane-associate HPK, which activates by phosphorylation the RR, which then activates expression of all operons necessary for bacteriocin synthesis, transport, and regulation [41, 42]. In these systems, the quorum sensing mechanism is not sufficient to maintain bacteriocin production in highly diluted cultures [36, 41–43], since concentration of the autoinducers (AIPs) or peptide pheromones produced is not sufficient to trigger full bacteriocin production, suggesting the involvement of other (environmental) factors.

Previously, we have shown that cell-to-cell contact with specific bacteria acts as environmental signals to switch on bacteriocin production in *Lactobacillus plantarum* NC8 through the activation of a quorum sensing mechanism involving the three component regulatory IPNC8-HKNC8-PlnD [24, 26, 44, 45]. This quorum sensing mechanism served to sense not only cell density of the NC8 population but, also, that of the competitor bacteria. This interspecies bacterial-bacterial phenomenon was shown to be widely distributed among *L. plantarum* strains [45, 46] and, in fact, the number of studies covering this subject has increased rapidly. Thus, to date, induction of bacteriocin production by specific bacteria has been described in *L. plantarum*, [26, 45–49], *Lactobacillus acidophilus* [43, 50], *Lactobacillus helveticus* [47], *Carnobacterium divergens* [51], *Leuconostoc citreum* [52] and *Enterococcus faecium* [47].

The analysis of the loci involved in GasE production showed the presence of a putative TCRS formed by the inducer peptide GaeP, the histidine protein kinase GaeK and the response regulator GaeR. The promoter sequences in front of the gene clusters involved in regulatory and biosynthesis of this bacteriocin has conserved regulatory sequences where the response regulator (GaeR) would bind to activate the expression of these regulated genes [36]. These putative regulatory elements are similar to those involved in quorum-sensing regulation of Blp bacteriocins in *Streptococcus thermophilus* [34]. As in other regulated class II bacteriocins, the AIP (GaeP) must be present in the environment at a certain threshold in order to be recognized by their cognate histidine kinase (GaeK), a process leading to the activation of the response regulator (GaeR) which, in turn, will activate expression of the proper regulatory operon (autoinduction mechanism) and the operon involved in biosynthesis of GasE [35, 36].

Based on phenotypic experiments and in the genetic data, we suggest that bacteriocin production by *L. gasseri* EV1461 is regulated by a quorum sensing mechanism involving the TCRS operon *gaeP*-*gaeK*-*gaeR*, and that specific bacteria induce bacteriocin production in *L. gasseri* EV1461 through the activation of such regulatory system, as it has been demonstrated in *L. plantarum* [44, 45]. Although the regulatory operon found in *L. gasseri* EV1461 (*gaeIP*-*gaeK*-*gaeR*) was very similar to those described in other *L. gasseri* strains, such as JV-V03 (GenBank acc. num. ACGO02000001), K7 [53] and LA158 [40], bacteriocin induction in these strains has not been described yet.

Bacterial vaginosis (BV) is the most common disorder of the female reproductive tract, characterized by the displacement of commensal vaginal lactobacilli and the overgrowth of mixed pathogenic bacterial populations [10, 11, 54, 55]. Interestingly, *L. gasseri* EV1461 has shown to possess inhibitory activity against the main pathogenic species of the bacterial vaginosis-associated bacteria (BVAB) [9–11], such *Atopobium vaginae*, *Gardnerella vaginalis*, *Porphyromonas gingivalis*, and *Prevotella bivia*.

## Conclusions

Presently, gassericin-producing *L. gasseri* strains had been isolated from either adult (*L. gasseri* SBT2055) or infant feces (*L. gasseri* K7, *L. gasseri* LF221, *L. gasseri* LA158); in contrast, *L. gasseri* EV1461 was isolated from the vagina of healthy woman. This origin and the ability to produce bacteriocins that inhibit the grown of pathogenic BVAB, may be an advantage for using *L. gasseri* EV1461 as a probiotic strain to fight and/or prevent bacterial infections as BV, since it could be better adapted to live and compete into the vaginal environment. The use of probiotic lactobacilli to prevent vaginal infection has a good rationale, and an excellent safety record, but so far only a few strains have been clinically proven to be effective, in particular to prevent BV [56].

More studies are required to elucidate the potential of *L. gasseri* EV1461 as a vaginal probiotic but its origin and its peculiar mechanism for bacteriocin production makes it a good candidate for such use in the future.

## Additional files

Additional file 1: Figure S1. Schematic representation of the locus for Gassericin E (GasE) production of *L. gasseri* EV1461 and comparison with the locus involved in the production of other similar *L. gasseri* bacteriocins. Incomplete orfs are indicated with an asterisk. In *L. gasseri* JV-03 and K7, the putative genes associated to a locus tag were shown in brackets. GeneBank accession numbers and references for these strains are: *L. gasseri* SBT2055 ([21]; AB029612); *L. gasseri* LA158 ([40]; AB710328); *L. gasseri* LF221 ([37]; AY297947); *L. gasseri* K7 ([38]; AY307382); *L. gasseri* K7 genome ([53]; ASRG02000002); *L. gasseri* JV-03 (Unpublished; ACGO02000001); *L. gasseri* G7 ([57]; KF724911). (PDF 183 kb) Additional file 2: Table S1. Comparison between deduced amino sequences of the Gassericin E locus against databases (GeneBank). (PDF 61 kb) Additional file 3: Figure S2. Bacteriocin activity of fractions obtained during first purification steps of Gassericin E through C_2_/C_18_ reverse-phase chromatography. The numbers in the plate indicate the fractions assayed. The black arrow indicates the putative complementary activity between different fractions, i.e, between fractions 16–17 and 24–25, which is most probably due to the diffusion and mixing of complementary active peptides. *L. gasseri* Lc9 was used as the indicator strain. (PDF 98 kb)

## Abbreviations

BV: bacterial vaginosis
GasE: gassericin E
CFSs: cell-free supernatants
PCR: polymerase chain reaction
BHI: brain heart Infusion
TSB: tryptic soy broth agar
RCA: reinforced clostridial agar broth medium
ETSA: enriched tryptic soy agar
RPC-FPLC: reverse-phase chromatography-fast protein liquid chromatography
SDS-PAGE: sodium dodecyl sulfate polyacrylamide gel electrophoresis
MALDI-TOF-MS: matrix-assisted laser desorption/ionization time of flight mass spectrometry
dNTPs: deoxynucleotides triphosphates
BLAST: basic local alignment search tool
Bac: bacteriocin
CFU: colony forming units
BU: bacteriocin units
ORFs: open reading frames
AIP: autoinducer peptide
HPK: histidine protein kinases
RR: response regulator
TCRS: three-component regulatory system
BVAB: bacterial vaginosis associated bacteria
