# Supplementary figures and images for: Purification and genetic characterization of gassericin E, a novel co-culture inducible bacteriocin from Lactobacillus gasseri EV1461 isolated from the vagina of a healthy woman
Source: BMC Microbiol. 2016 Mar 12;16:37. doi: 10.1186/s12866-016-0663-1 (PMC4788914; doi:10.1186/s12866-016-0663-1)

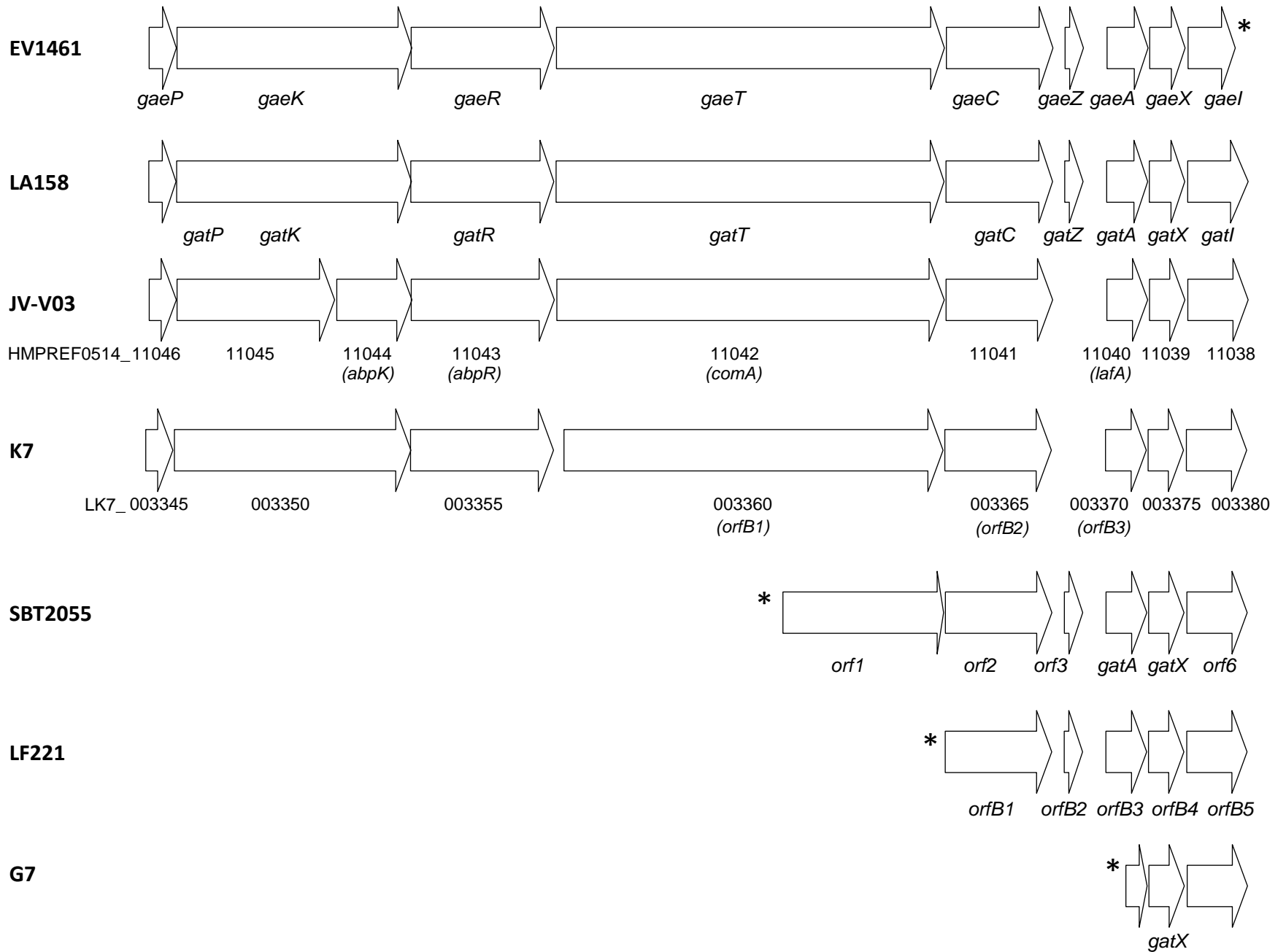

Supplement: Additional file 1: Figure S1. — Schematic representation of the locus for Gassericin E (GasE) production of L. gasseri EV1461 and comparison with the locus involved in the production of other similar L. gasseri bacteriocins. Incomplete orfs are indicated with an asterisk. In L. gasseri JV-03 and K7, the putative genes associated to a locus tag were shown in brackets. GeneBank accession numbers and references for these strains are: L. gasseri SBT2055 ([21]; AB029612); L. gasseri LA158 ([40]; AB710328); L. gasseri LF221 ([37]; AY297947); L. gasseri K7 ([38]; AY307382); L. gasseri K7 genome ([53]; ASRG02000002); L. gasseri JV-03 (Unpublished; ACGO02000001); L. gasseri G7 ([57]; KF724911). (PDF 183 kb) [file 12866_2016_663_MOESM1_ESM.pdf]

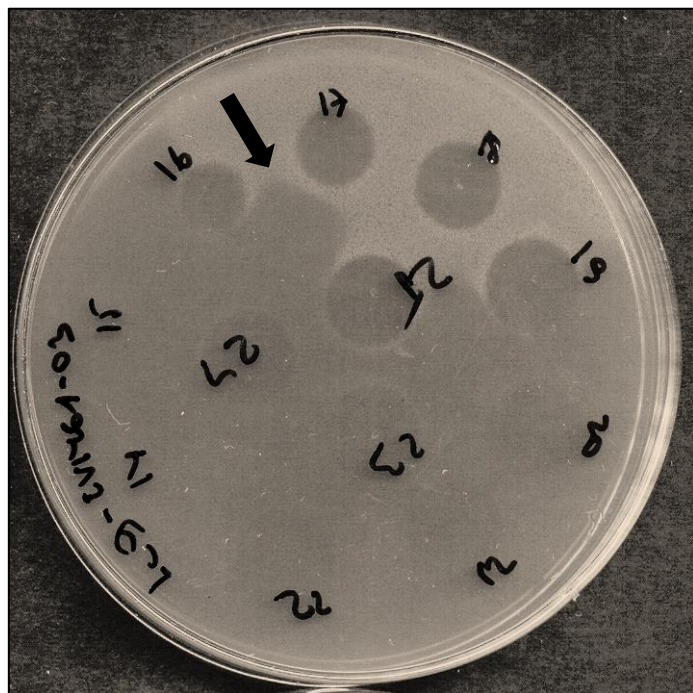

Figure S2

Supplement: Additional file 3: Figure S2. — Bacteriocin activity of fractions obtained during first purification steps of Gassericin E through C2/C18 reverse-phase chromatography. The numbers in the plate indicate the fractions assayed. The black arrow indicates the putative complementary activity between different fractions, i.e, between fractions 16–17 and 24–25, which is most probably due to the diffusion and mixing of complementary active peptides. L. gasseri Lc9 was used as the indicator strain. (PDF 98 kb) [file 12866_2016_663_MOESM3_ESM.pdf]
