# Supplementary material for: Purification and genetic characterization of gassericin E, a novel co-culture inducible bacteriocin from Lactobacillus gasseri EV1461 isolated from the vagina of a healthy woman
Source: BMC Microbiol. 2016 Mar 12;16:37. doi: 10.1186/s12866-016-0663-1 (PMC4788914; doi:10.1186/s12866-016-0663-1)
Supplement: Additional file 2: Table S1. — Comparison between deduced amino sequences of the Gassericin E locus against databases (GeneBank). (PDF 61 kb) [file 12866_2016_663_MOESM2_ESM.pdf]

Table S1

Gene (Protein; aas)	Name (aas)	Gene/Locus Tag	%Id[2]	Comments[3]
**Bacteriocin**				
gaeA (Gassericin E; 75)	Gassericin T (75)	*gatA*	99 (74/75)	Bacteriocin Gassericin T (*L. gasseri* SBT2055)
	Gassericin T (75)	*gatA*	99 (74/75)	Prepeptide Gassericin T (*L. gasseri* LA158)
	Acd221β (75)	*orfB3*	99 (74/75)	Putative complement factor (*L. gasseri* LF221)
	GasK7B_α (75)	*orfB3*	99 (74/75)	Putative gassericin K7 B complemental factor (*L. gasseri* K7)
	LafA (75)	*lafA*/HMPREF0514_11040	97 (73/75)	Bacteriocin lactacin-F subunit LafA (*L. gasseri* JV-03)
	GasK7B_α (75)	LK7_003370	97 (73/75)	Putative gassericin K7 B complemental factor (*L. gasseri* K7)
gaeX (GaeX; 65)	GatX (65)	*gatX*	100 (65/65)	Putative bacteriocin complemental factor (*L. gasseri* SBT2055)
	-	HMPREF0514_11039	100 (65/65)	Bacteriocin-type signal sequence (*L. gasseri* JV-03)
	GatX (65)	*gatX*	100 (65/65)	Bacteriocin (*L. gasseri* LA158)
	GasK7B_β	LK7_003375	100 (65/65)	Gassericin K7 B (*L. gasseri* K7)
	GatX (65)	*gatX*	100 (65/65)	Bacteriocin (*L. gasseri* G7)
	Acd221B (65)	-	98 (64/65)	Acidocin LF221B (*L. gasseri* LF221)
	GasK7B_β	-	98 (64/65)	Gassericin K7 B (*L. gasseri* K7)
gaeI (GaeI; 88)[1]	ORF-6 (112)	*orf-6*	100 (88/112)	Putative immunity protein (*L. gasseri* SBT2055)
	GatI (112)	*gatI*	100 (88/112)	Putative immunity protein (*L. gasseri* LA158)
	Aci221B (112)	-	100 (88/112)	Putative immunity protein (*L. gasseri* LF221)
	GaiK7B(112)	-	100 (88/112)	Putative gassericin K7 B immunity protein (*L. gasseri* K7)
	- (112)	LK7_003380	100 (88/112)	Putative gassericin K7 B immunity protein (*L. gasseri* K7)
	- (112)	HMPREF0514_11038	100 (88/112)	Hypothetical protein (*L. gasseri* JV-03)
	- (99)[1]	-	100 (88/99)	Putative immunity protein (*L. gasseri* G7) AHE41140 (Stoyancheva)
**Regulation**				
gaeP (GaeP; 50)	- (50)	HMPREF0514_11046	100 (50/50)	Hypothetical protein (*L. gasseri* JV-03)
	GatP (50)	*gatP*	100 (50/50)	Putative pheromone peptide (*L. gasseri* LA158)
	- (50)	LK7_003345	100 (50/50)	Lactacin F two-component system inducer peptide precursor (*L. gasseri* K7)
gaeK (GaeK; 435)	GatK (435)	*gatK*	99 (430/435)	Putative Histidine Kinase (*L. gasseri* LA158)
	- (437)	LK7_003350	98 (426/435)	Histidine Kinase (*L. gasseri* K7)
	- (292)	HMPREF0514_11045	98 (279/284)	Hypothetical protein (*L. gasseri* JV-V03)
gaeR (GaeR;265)	- (265)	*abpR*/HMPREF0514_11043	100 (265/265)	LytTr DNA-binding domain protein (*L. gasseri* JV-V03)
	GatR (265)	*gatR*	100 (265/265)	Putative response regulator (*L. gasseri* LA158)
	- (265)	LK7_003355	100 (265/265)	Chemotaxis protein CheY (*L. gasseri* K7)
**Transport**				
gaeT (GaeT; 719)	- (719)	*comA*/ HMPREF0514_11042	99 (718/719)	ABC-type bacteriocin transporter (*L. gasseri* JV-V03)
	- (719)	LK7_003360	99 (718/719)	Peptide ABC transporter ATP-binding protein (*L. gasseri* K7)
	GatT (719)	*gatT*	99 (718/719)	Putative ABC-transporter (*L. gasseri* LA158)
	ORFB1 (465)[1]	*orfB1*	99 (465/465)	Putative ABC-transporter (*L. gasseri* K7)
gaeC (GaeC; 197)	ORFB2 (197)	*orfB2*	99 (195/197)	Putative gassericin K7 B accessory protein (*L. gasseri* K7)
	- (197)	HMPREF0514_11041	99 (195/197)	Hypothetical protein (*L. gasseri* JV-V03)
	- (197)	LK7_003365	99 (195/197)	Putative gassericin K7 B accessory protein (*L. gasseri* K7)
	ORF2 (197)	*orf-2*	98 (194/197)	Hypothetical protein (*L. gasseri* SBT2055)
	GatC (197)	*gatC*	98 (194/197)	Hypothetical protein (*L. gasseri* LA158)
	ORFB1 (188)	*orfB1*	99 (186/188)	Unknown, partial (*L. gasseri* LF221)
**Unknown**				
gaeZ (GaeZ; 33)	ORF-3 (33)	*orf-3*	100 (33/33)	Putative expression regulator for Gassericin T (*L. gasseri* SBT2055)
	ORFB2 (33)	*orfB2*	100 (33/33)	Unknown (*L. gasseri* LF221)
	GatZ	*gatZ*	100 (33/33)	Unknown (*L. gasseri* LA158)
	ORFhip	*orfBhip*	100 (33/33)	Hypothetical protein (*L. gasseri* K7)

[1] Partial orf. [2] %Id: percent of amino acids identity. The number of identical aminoacids with respect to the total lenght of the amino acids sequence compared is shown in brackets.
[3] GeneBank accession numbers and references for these strains are: *L. gasseri* SBT2055 (21; AB029612); *L. gasseri* LA158 (40; AB710328); *L. gasseri* LF221 (37; AY297947); *L. gasseri* K7 (38; AY307382); *L. gasseri* K7 genome (57; ASRG02000002); *L. gasseri* JV-03 (Unpublished; ACGO02000001); *L. gasseri* G7 (51; KF724911).
